# Ventricular myocardium development and the role of connexins in the human fetal heart

**DOI:** 10.1038/s41598-017-11129-9

**Published:** 2017-09-25

**Authors:** Eleftheria Pervolaraki, James Dachtler, Richard A. Anderson, Arun V. Holden

**Affiliations:** 1 School of Biomedical Sciences, University of Leeds, Leeds, LS2 9JT UK; 2 Department of Psychology, Durham University, Durham, DH1 3LE UK; 3 MRC Centre for Reproductive Health, University of Edinburgh, Edinburgh, EH16 4TJ UK

## Abstract

The developmental timeline of the human heart remains elusive. The heart takes on its characteristic four chambered appearance by ~56 days gestational age (DGA). However, owing to the complexities (both technical and logistical) of exploring development *in utero*, we understand little of how the ventricular walls develop. To address this, we employed diffusion tensor magnetic resonance imaging to explore the architecture and tissue organization of the developing heart aged 95–143 DGA. We show that fractional anisotropy increases (from ~0.1 to ~0.5), diffusion coefficients decrease (from ~1 × 10^−3^mm^2^/sec to ~0.4 × 10^−3^mm^2^/sec), and fiber paths, extracted by tractography, increase linearly with gestation, indicative of the increasing organization of the ventricular myocytes. By 143 DGA, the developing heart has the classical helical organization observed in mature mammalian tissue. This was accompanied by an increase in connexin 43 and connexin 40 expression levels, suggesting their role in the development of the ventricular conduction system and that electrical propagation across the heart is facilitated in later gestation. Our findings highlight a key developmental window for the structural organization of the fetal heart.

## Introduction

The known structure of the adult human heart (with its helical fiber angles and organization) can only be identified during the later stages of gestation^1^, with the early fetal human heart not exhibiting this helical organization and structure^1^ as described in the adult^2^. Initially the heart develops from an early tube at ~20 days of gestational age (DGA)^3^ with the four-chambered structure being visible after ~56 DGA^4^.

The application of imaging technologies, such as diffusion tensor magnetic resonance imaging (DT-MRI), has previously been used to explore the detailed structure of the fetal human heart^1^. DT-MRI derived datasets can be used to describe the structure and organization of the human heart during gestation depending on its anisotropic and orthotropic properties. DT-MRI analysis has shown that the human fetal heart is highly isotropic in early gestational stages^1^. This means that diffusion moves equally in all directions within the tissue, as illustrated by the “chaotic” and non-organized cardiac structure we previously presented^1^. As the heart develops, we have previously demonstrated that its anisotropy increases with diffusion moving preferentially in one prominent direction, after ~140 DGA, creating a helical organization of the cardiac fibers with smooth angle changes^1^. These diffusion properties can be used to create computational models and algorithms able of computing the fiber tracks within a heart during development, as the ones presented in this paper. Fiber tracking, or tractography, is an MRI-based visualization technique for computing the location, orientation and anisotropy of tracts around any given tissue.

Previously, we demonstrated that the smooth helical organization (observed in the adult heart with a distinct ~120° transmural slope) can only be seen in the fetal heart from ~140 DGA^1^. This helical structure and tissue organization are important for normal cardiac function. The orientation of cardiac myofibers within that helical organization, in the adult heart and the fetal human heart in later stages of gestation, is important for propagation of ventricular excitation^5^ and allows coordinated contraction of the ventricular tissue^6,7^. During diastole and systole, the ventricular wall thickness decreases as ventricular volume increases^8^. This is facilitated by the structure of the ventricular myocardium, which consists of plate like structures running within the helical organization^9^. Any changes to this structure can lead to fibrosis and remodeling of the helical organization, affecting normal cardiac function^10^. Hence, understanding the rate of development of this helical structure and the mechanisms involved, in the human, may elucidate important developmental processes crucial for normal cardiac function, or even pathology.

Knowledge of the structure of the fetal human heart, derived from DT-MRI^1,11^, alone does not provide a complete understanding of human heart development. The exploration of the molecular mechanisms necessary for propagation of excitation through the ventricular tissue and their developmental regulation will help elucidate the process of how ventricular fiber organization occurs. Thus, studying the expression of gap junctions found in the ventricular myocardium during fetal development is necessary, as it is known that gap junctions between myocytes facilitate the propagation of excitation within the myocardium^12,13^. Gap junctions are channels called connexons which are hexamers of proteins called connexins. The most abundant connexin found in the ventricular myocardium is connexin 43 (Cx43). Its expression and distribution throughout the ventricular tissue plays a crucial role in the development of the ventricular structure and its remodeling^14^. Thus, Cx43 expression during early fetal life could facilitate ventricular structural organization by enabling myocyte-to-myocyte electrical communication, therefore promoting propagation properties within the developing heart.

Cardiomyocytes co-express connexin 43 (Cx43) with connexin 40 (Cx40), another gap junction expressed in the mammalian heart. Their pattern of co-expression is thought to be important for the maintenance of normal heart rate^15^. Cx40 is the main gap junction protein found in the ventricular conduction system (the His bundle and the upper parts of the bundle branches), and it is thought to play a developmental role in the appearance of Purkinje fibers during mammalian gestation^16^.

In this study, we quantify the developmental changes of myocardial architecture of *ex vivo* fetal hearts by DT-MRI. DT-MRI has been validated as providing a measure of cardiomyocyte orientation^1,17–19^ and an index of the helical structure of the ventricular wall^1,20,21^, and has provided measurements of myocardial fiber and lamellar orientations in mammalian ventricular myocardium^1,20,22^. However, we currently lack an understanding of how the detailed microstructure of the ventricular myocardium develops over gestation. Previous studies have used a very limited sample size^1,11^, from which statistically robust conclusions cannot be made. Herein, we use DT-MRI protocols (with a cubic voxel size of 100 µm) to quantify the development of the ventricular wall, by means of diffusion properties, in 23 fetal human hearts aged from 95 to 143 DGA, thereby being the first study to provide a complete developmental timeline of the human ventricular development across the first and second trimester. We have also quantified the expression of Cx43 and Cx40 in developing human hearts from 67 to 136 DGA. We correlate the increased expression of these two connexins (Cx43 and Cx40) with the increased structural organization of computed cardiac fiber paths observed with gestational age.

## Results

### Ventricular wall maturation reflects increases in fractional anisotropy (FA) and decreases in apparent diffusion coefficient (ADC)

Diffusion properties can be used to calculate the development of the ventricular wall. We have previously shown that by ~140 DGA, the ventricular wall (as measured by FA) has become more anisotropic^1^ and largely resembles mature tissue. A related measure, ADC, has yet to be applied to human fetal cardiac tissue. ADC quantifies the diffusion rate within a tissue, and is thought to be a more biologically-relevant quantification of DT-MRI datasets. In order to provide a full developmental timeline of ventricular wall maturation, including left/right comparisons, 23 hearts were scanned and FA and ADC quantified.

**Figure 1:**
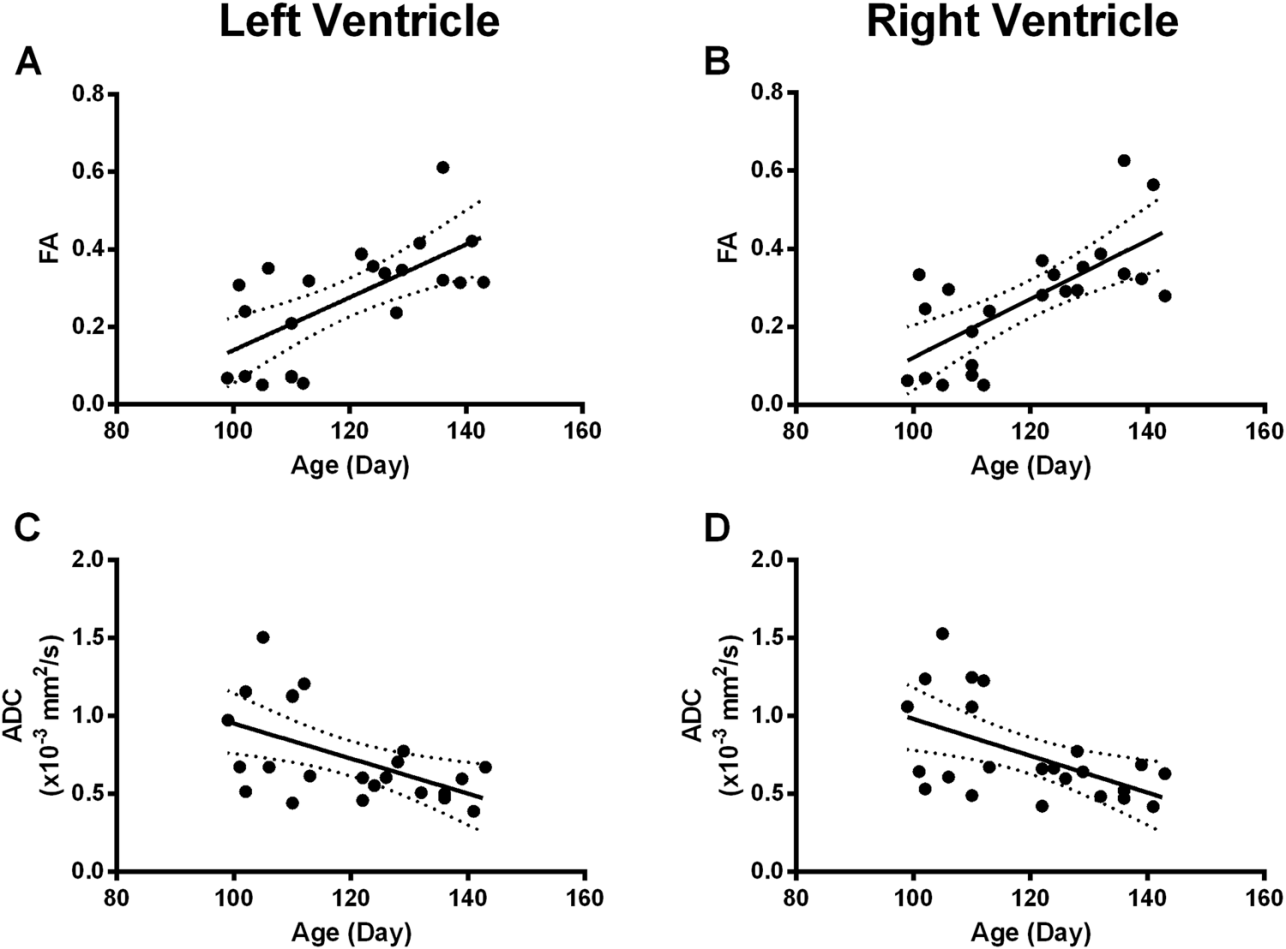
Development of ventricular wall organization during gestation. Fractional anisotropy (**A** and **B**) and apparent diffusion coefficient (**C** and **D**) for the left (**A** and **C**) and right (**B** and **D**) ventricles for human fetal hearts aged 95–143 DGA. Fractional anisotropy (FA) is illustrated as a scalar value from 0 to 1. FA significantly increases within the left ventricle (**A**: p = 0.0007, R^2^ = 0.43) and the right ventricle (**B**: p = 0.0002, R^2^ = 0.49) with gestational age. Apparent diffusion coefficient (ADC) quantifies the average diffusion within the wall of each ventricle. It was found to significantly decrease in the left (**C**: p = 0.009, R^2^ = 0.29) and right (**D**: p = 0.008, R^2^ = 0.29) ventricle and with gestational age.

FA was estimated from DT-MRI datasets for the segmented ventricles (right and left) and increased with gestation from <0.1 to 0.6 as gestation approaches the third trimester (Fig. 1, panels A and B). We have identified a correlation of ventricular FA to gestational age, indicative of an increase in tissue organization. Linear regression revealed statistical significance in the increase of ventricular FA (F_(1,21)_ = 20.22, p = 0.0002, R^2^ = 0.49 for the right ventricle; F_(1,21)_ = 15.92, p = 0.0007, R^2^ = 0.43 for the left ventricle). There was no significant difference between the regression slopes for both ventricles (F_(1,42)_<1), suggesting a similar rate of right/left ventricular development.

Ventricular ADC was also measured and decreased with age from approximately 1 × 10^−3^ mm^2^/sec to 0.4 × 10^−3^ mm^2^/sec between 95 and 143 DGA (Fig. 1, panels C and D). Regional ADC (illustrated for both segmented ventricles in Fig. 1) significantly decreased with age (F_(1,21)_ = 8.58, p = 0.008, R^2^ = 0.29 for the right ventricle and F_(1,21)_ = 8.38, p = 0.009, R^2^ = 0.29 for the left ventricle). The ADC data suggest that there is more variance at younger developmental stages, linked to the diffusion within the radial direction (measured by changes in λ_2_ and λ_3_). As the complexity of the tissue increases, diffusion becomes more restricted and as a result ADC reduces.

### Tractography reveals the development of computed cardiac fibers

Tractography analysis of DT-MRI derived datasets is more commonly associated with exploring fiber tracts within the brain, although recently their application has extended to the heart. Tractography works by computing the preferred orientation of subsequent voxels; those that follow a connected ‘path’ are likely anatomically linked. We hypothesized that tractography would be particularly useful for the study of development; fibers that were not fully developed would show reduced fiber complexity, length and density.

**Figure 2:**
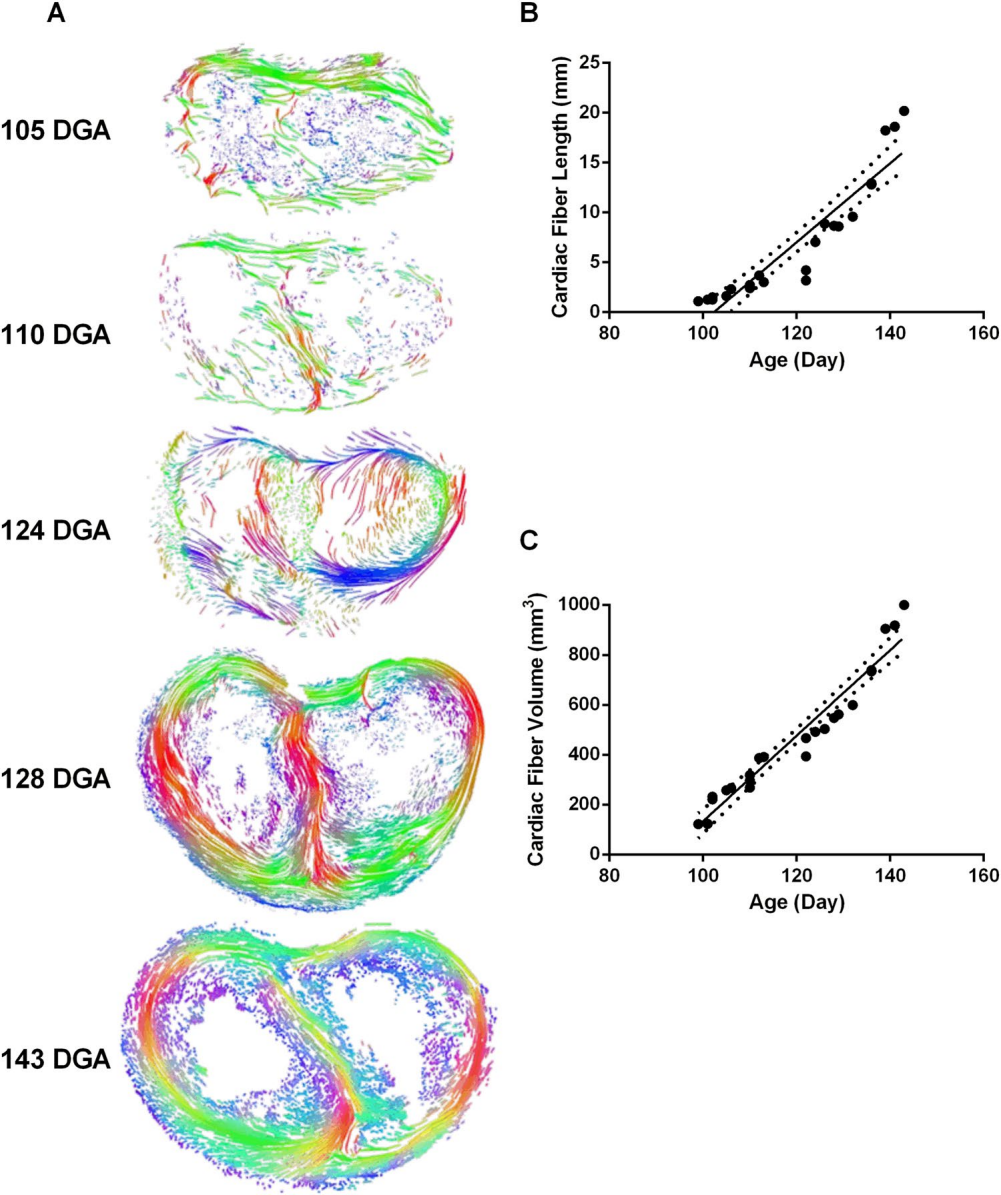
Tractography of ventricular wall fibers during gestation. (**A**) Cardiac fibers computed from DT-MRI derived ventricular fiber architecture, for fetal hearts aged 105, 110, 124, 128 and 143DGA, are interpreted visually with a coloring scheme based on the curve features of local tangent directions. The length (**B**) and volume (**C**) of the computed ventricular cardiac fibers for human fetal hearts were found to significantly correlate with gestation (p < 0.0001 for both length and volume; R^2^ = 0.87 for length and R^2^ = 0.94 for volume).

To test whether the ventricular wall shows developmental regulation of fiber tracts, we computed the architecture of cardiac fiber paths by tractography (Fig. 2, panel A). We observed an increase in the length and density of the cardiac fiber paths in the ventricular myocardium during development. Linear regression (Fig. 2, panels B and C) demonstrated significant correlations for both length (F_(1,21)_ = 138.7, p < 0.0001, R^2^ = 0.87) and volume (F_(1,21)_ = 308, p < 0.0001, R^2^ = 0.94). Together, this confirms that within the first trimester, the ventricular wall has yet to take on any complex and regular features, possibly as the myocardium is still dis-organized. This is particularly evident between 124 and 128 DGA, where interventricular septum and ventricular walls rapidly become ordered and have distinct fiber tracts (Fig. 2, panel A).

**Figure 3:**
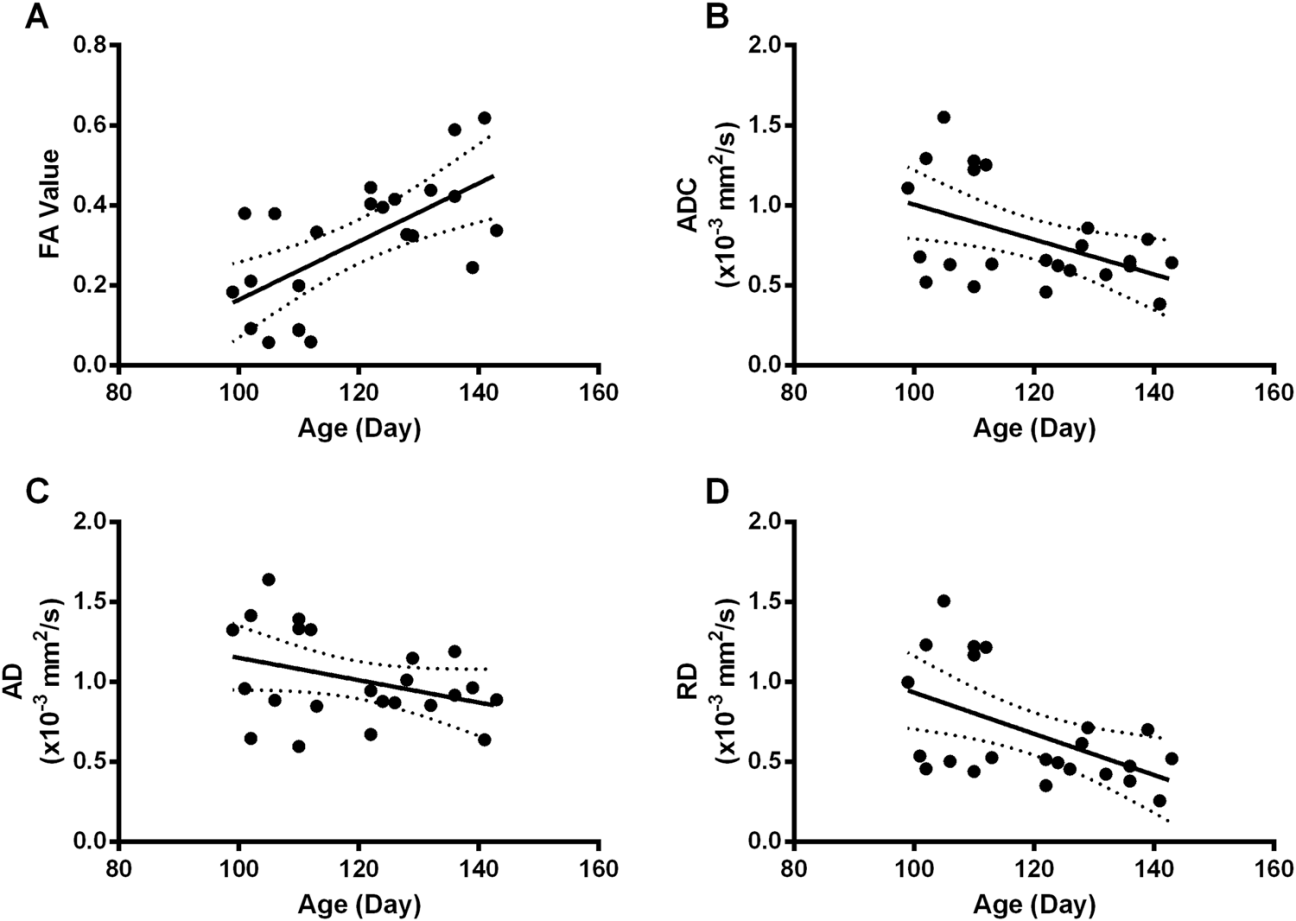
Quantification of ventricular wall cardiac fibers during gestation. (**A**) The fractional anisotropy (FA) of the computed cardiac fibers, derived from tractography performed on DT-MRI datasets, was found to significantly increase with gestational age (p = 0.0009, R^2^ = 0.41). (**B**) Apparent diffusion coefficient (ADC) was also found to be significantly correlated with age (p = 0.0196, R^2^ = 0.23). To further identify the extend of these changes, we investigated diffusions in the primary (**C**) and tertiary (**D**) orientation. We found that only the radial diffusion (RD; **D**) was significantly correlated with gestation (p = 0.011, R^2^ = 0.27).

Quantitative analysis of the diffusion properties of the cardiac fiber paths revealed a consistent increase in the FA of ventricular fibers and a decrease in ADC with gestational age (Fig. 3, panels A and B). Linear regression demonstrated significant correlations with age for both FA (F_(1,21)_ = 14.81, p = 0.0009, R^2^ = 0.41) and ADC (F_(1,21)_ = 6.37, p = 0.0196, R^2^ = 0.23). Given ADC measures diffusion in three directions (λ_1_, λ_2_ and λ_3_), we sought to test whether the decrease in ADC was being driven predominately by diffusion in the axial (λ_1_) or the radial orientation (λ_2_ and λ_3_) by characterization of axial diffusion (AD; primary) and radial diffusion (RD; radial) (as in equation [2]) components (Fig. 3, panels C and D). Changes in AD and/or RD suggest whether development is dependent upon fiber path orientation in the axial direction (e.g. directly along a cardiac fiber) or the radial, which could be due to volume of the fiber or by branching effects, respectively. RD was found to decrease with age. Linear regression analysis revealed a non-significant decrease in AD with gestational age (F_(1,21)_ = 2.99, p = 0.099) whereas RD was significantly negatively correlated (F_(1,21)_ = 7.85, p = 0.011, R^2^ = 0.27). This potentially reflects increasing organization of the ventricular tissue with development, given there is less ‘branching’ of myocytes owing to their more regular orientation.

### Connexin 43 (Cx43) and Connexin 40 (Cx40) expression levels increase with development

**Figure 4:**
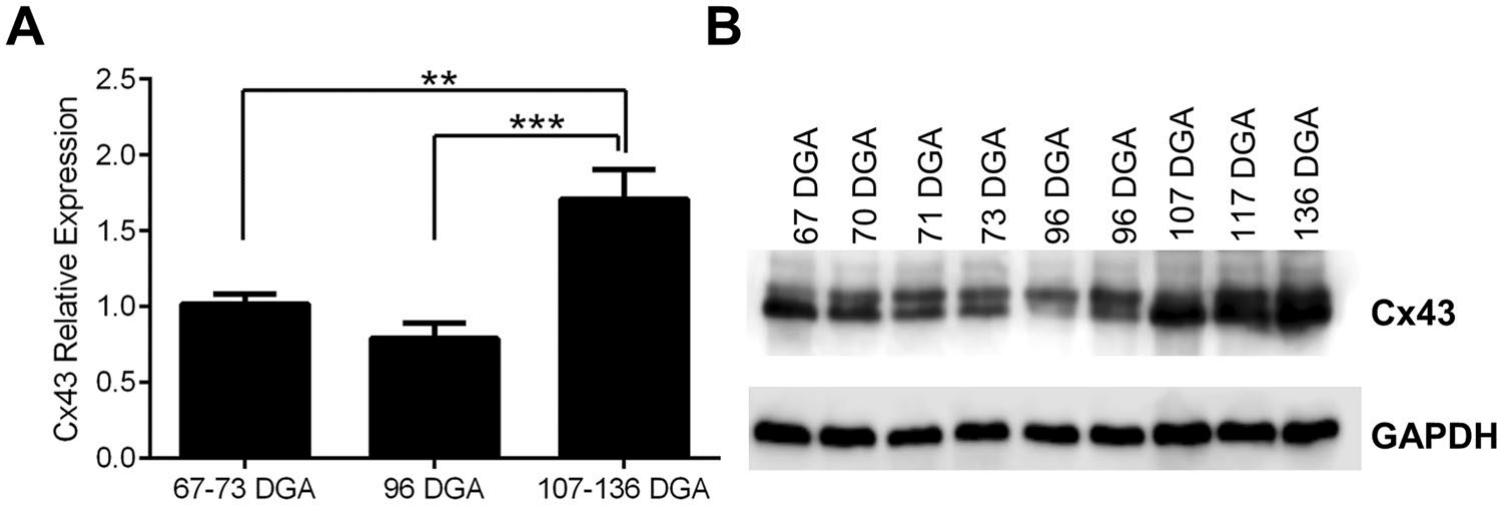
Connexin 43 expression, relative to GAPDH, in human fetal hearts aged 67–137 DGA. (**A**) Cx43 expression is significantly increased in the mid-second trimester (107–136 DGA) when compared to 67–73 DGA and 96 DGA. (**B**) Example Western blot of Cx43 expression across gestation, and the GAPDH loading control, which did not demonstrate developmental regulation. (**p = 0.001 and ***p = 0.0004).

The conduction of electrical signaling within the ventricles is dependent upon gap junction signaling between myocytes, of which the most abundant is Cx43. Deletion of Cx43 is known to severely alter the geometric anatomy of, particularly, the right ventricle^23^. We predicted that electrical coupling of myocytes via Cx43 gap junctions could facilitate myocyte organization via an activity-dependent process. Hence, increasing Cx43 expression could lead to greater synchronized activity, thus explaining the developmentally-regulated organization of the fiber paths of the developing heart. To test this, we examined protein extracted from fetal hearts ranging from 67–136 DGA, equivalent to the first and mid-second trimester (Fig. 4). With increasing development, Cx43 expression significantly increased (one-way ANOVA, F_(2,24)_ = 12.45, p = 0.0002). Cx43 expression was significantly greater between 67–73 DGA and 107–136 DGA (p = 0.001), and between 96 DGA and 107–136 DGA (p = 0.0004), highlighting the potential role of Cx43 in fetal heart maturation via gap junction-mediated cellular signaling pathways.

**Figure 5:**
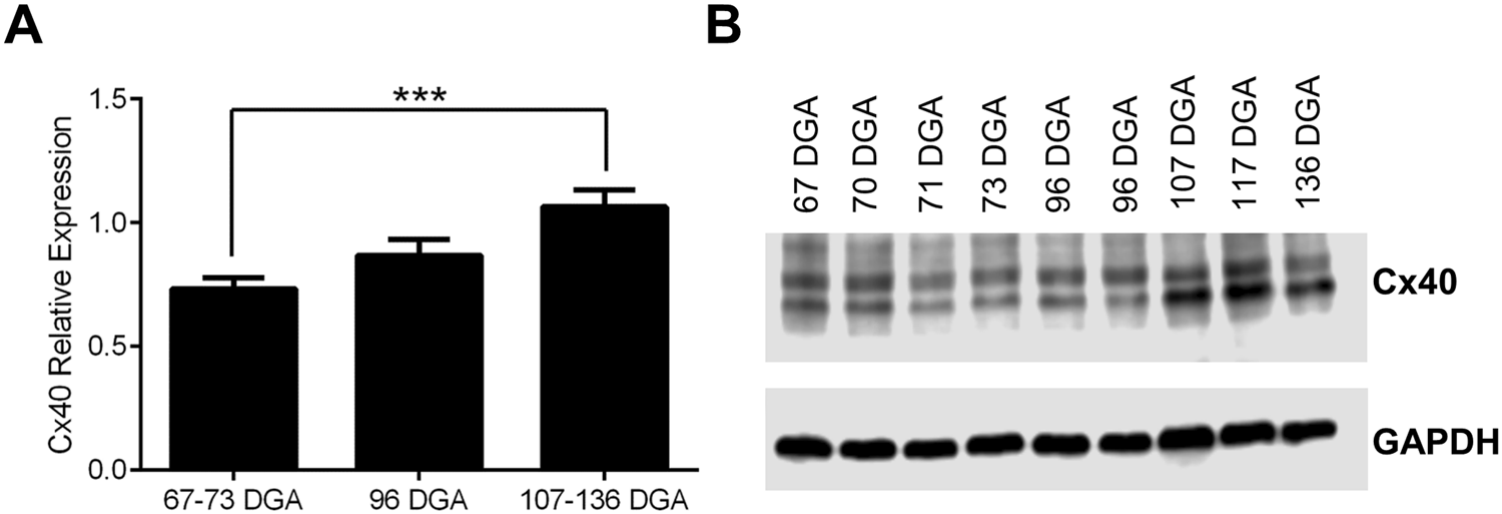
Connexin 40 expression, relative to GAPDH, in human fetal hearts aged 67 to 137 DGA. (**A**) Cx40 expression is significantly increased in the mid-second trimester (107–136 DGA) compared to the first trimer (67–73 DGA). (**B**) Example Western blot of Cx40 expression across development, and the GAPDH loading control, which did not demonstrate developmental regulation. ***p = 0.001.

It has also been shown that expression of Cx40^24–26^ has a role in the formation of the mammalian ventricular conduction system during development and its loss results in cardiac abnormalities^27^. Although the absence of Cx40 does not affect the gross structure of the heart, it results in conduction deficits with a reduced cardiac conduction velocity^27,28^. We therefore quantified Cx40 expression in developing hearts aged 67–136 DGA (Fig. 5). Cx40 increased throughout development, with significantly greater expression between ages 107–136 DGA (mid-second trimester) compared to 67–73 DGA (early development in the first trimester) (one-way ANOVA, F_(1,32)_ = 9.43, p = 0.001; 67–73 vs. 107–136 DGA p = 0.0006).

## Discussion

Within the current study, we have employed high-resolution diffusion tensor magnetic resonance imaging (DT-MRI) to image the human heart during development (95–143 days gestational age (DGA)) and visualize computed cardiac fiber path lines (Fig. 2) of ventricular myocardium with quantitative measurements of its anisotropy (such as fractional anisotropy (FA), apparent diffusion coefficient (ADC), radial diffusion (RD) and axial diffusion (AD)).

DT-MRI has developed into a useful tool for the investigation of changes within tissue microstructure^29,30^. DT-MRI derived datasets can provide directional information of tissue fibers and have been used to predominately map connections within the brain^31^ and heart^32^. Such studies have combined DT-MRI with tractography to map normal brain and heart physiology (creating population-averaged atlases) and to examine disease states. Availability of detailed atlases^32,33^ of the structure of complex organs with 3D visualizations of their fibers is important and necessary for guiding the interpretation of clinical data and aiding medical diagnosis and treatment. As yet these approaches have not been applied to the developing human heart, during gestation.

This is a first preliminary application of DT-MRI derived tractography using a sufficient number of human fetal hearts (compared to ref.^1^ and ref.^11^) to allow statistical analysis of observed developmental changes. Our study uses indices, such as ADC, AD and RD, to quantify the developing myocardial tissue architecture and relate these changes to an increase of Connexin 43 (Cx43) expression, the main connexin protein found in the ventricular myocardium, and Connexin 40 (Cx40), the main connexin protein expressed in the ventricular conduction system.

Using specialized imaging protocols and visualization software we have been able to quantify an increase of ventricular FA and a decrease of ventricular ADC in human fetal hearts aged from 95 DGA to 143 DGA. The FA and ADC changes that we observed are presumably due to developmental changes in the myocyte growth and cellular organization facilitated by the increase in Cx43 and Cx40 expression levels (Figs 4 and 5). The increase in ventricular FA in the human fetal hearts from 95 DGA to 143 DGA can also be explained by changes in the radial diffusion (RD) coefficient, which is the average of the secondary and tertiary eigenvalues. This means changes in higher level tissue organization, or the development of sheet-like structures^2^, occurs in the early second trimester in the human.

The development of greater tissue complexity and more tortuous diffusion paths (manifested with changes in myocyte shape, the development of more confined extracellular spaces and an increase in gap junction density) also has an effect on the ADC, which was found to decrease with gestational age. This would account for the changes of the radial diffusion (RD) we observed, which occurs perpendicularly to the principal diffusion and it is dependent on the secondary and tertiary eigenvectors.

The development of more complex spaces and diffusion paths lead to a reduction of the ease of diffusion in ventricular myocardial tissue, possibly due to an increase in cellular density^34^. Our tractographic analysis has revealed an increase in the myocardial organization, evidenced by an increase in the density of fiber paths and their length across gestation (Fig. 2). Their highly organized architecture and their increased volume (Fig. 2) are a result of possible proliferative activity within the fetal ventricular myocardium^35,36^.

These changes can also be related to the increased expression levels of Cx43 (Fig. 4) and Cx40 (Fig. 5) that we observed, which have a role in the development of the working myocardium and ventricular conduction system respectively. Our data show that the development of the helical organization of the myocardial tissue architecture (Fig. 2) follows after the development of anisotropy within the human fetal heart and it correlates with the increased expression of Cx43 and Cx40. An increase in the expression of connexin proteins might be indicative of normal gap junction development for effective cell-cell communication and coupling leading to an organized contraction during gestation. One limitation of the current study is that we have only examined the protein expression changes of Cx40 and Cx43. It is likely that during gestation, a great number of genes/proteins become up or downregulated. Whether Cx40 and Cx43 can fully explain the maturation of the ventricular wall is unclear by this study alone. However, it will be interesting for future studies to examine the genomic, transcriptomic and proteomic maturation of the fetal heart to find which genes are critical for structural development.

In this study, we are showing that the known helical architecture of the heart is, indeed, formed during human fetal development and any developmental abnormalities may impact on the later structure of the heart during childhood and adulthood. Impaired development of the fiber architecture and geometry may lead to early onset of cardiac pathologies^37,38^ characterized by dysfunctional laminar organization of sheets.

The organization and orientation of the helical myocardial fibers can be altered in myopathies or other diseased states (e.g. pulmonary hypertension^39^ or hypertrophy^40^). These structural changes alter the pattern of propagation, which may lead to arrhythmias^41^. Once the physiologically normal cardiac organization (Fig. 2) is mapped quantitatively, we are in a position to be able to identify disarrayed fetal myocardial development that may result in ventricular non-compaction/spongiform cardiomyopathy or mimic the processes of diabetic cardiomyopathy and dilated cardiomyopathy. We anticipate that abnormalities in ventricular development (such as congenital heart anomalies) could be associated with quantitative changes in transmural angle and global helical architecture of ventricular myocytes as well as altered expression and localization of key proteins. Quantitative characterization of these changes should enable the interpretation of the relationship between ventricular structure and the development of pathologies.

**Figure 6:**
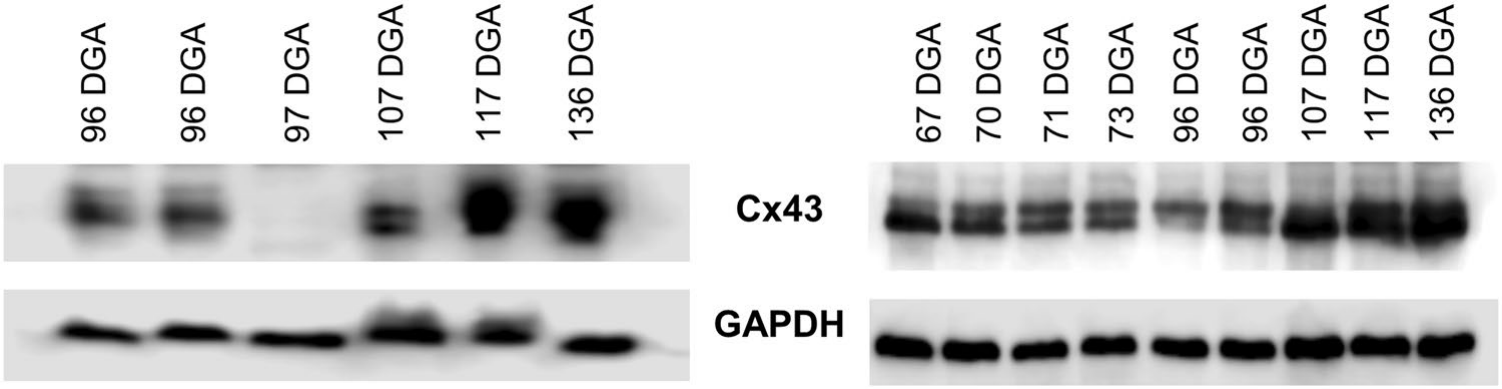
The absence of Connexin 43 expression in one human fetal heart aged 97 DGA. In the process of testing Cx43 expression in the fetal heart samples, we discovered that one heart aged 97 DGA lacked any detectable Cx43 expression (left, top, sample 3). We confirmed this across three separate gels. Despite the lack of Cx43, the GAPDH loading control produced a clear band (left, bottom). At comparative ages (e.g. 73–107 DGA) Cx43 expression was clearly detectable in all other samples (right, top; blots from Fig. 4).

During the process of estimating the expression changes of Cx43 during gestation, we identified one heart aged 97 DGA which did not express the protein (Fig. 6). This heart, as with every other sample, was clinically assessed to be normal upon extraction. Indeed, the weight of the heart was broadly similar to other age matched hearts (96 DGA: 0.183 g vs 97 DGA: 0.178 g). Although we were unable to image this heart, we hypothesize that the fetus’ myocardium was not developing as expected. It has been demonstrated that decreased levels or altered trafficking of Cx43 can lead to decreased cell-cell coupling in the ventricular myocardium which in turn may affect the process of septation and exhibit reduced propagation velocity^42^. We can only postulate about the clinical pathology that may have accompanied this heart. However, given that this sample was exhibiting a complete loss of Cx43 expression (and not just a reduction), we believe that at full term multiple abnormalities would have been present. Although this finding was serendipitous, it does represent 9% of our total sample, which may highlight new and important research avenues for a role of connexins in normal heart development.

Effective cardiac conduction is aided by tissue geometry (depending on cell density, size, and shape) and the distribution of gap junctions. Connexin 40 is expressed in the ventricular conduction system and the fast-conducting atrial myocardium. Later in development of the mammalian heart, Cx40 can be found expressed in the trabecular network and the Purkinje fibers network^43^. Previous studies have demonstrated that conduction pathologies (such as right bundle branch block or slower conduction velocity) are present in Cx40 deficient mice^44,45^. Our study revealed a gradual increase in the expression levels of Cx40 during the development of the human fetal heart. Our preliminary study may demonstrate the requirement for Cx40 at early human ventricular development but the role of other proteins and their expression profiles should be further investigated.

In summary, this is the first study that structural data (acquired from DT-MRI) and protein expression data were combined with computational modelling in an effort to map human heart development. We have used DT-MRI and visualization tools to characterize and quantify the development of the structure and organization of the human fetal ventricular myocardium. We have identified the development of the organization of the ventricular streamlines (cardiac fibers) and we have shown an increase in their length and density relative to gestational age. We have also quantified the expression of Cx43 and Cx40 and its increase during gestation in the human fetal heart. Although preliminary, the current study presents an advancement in our understanding of fetal cardiac development, and future studies will expand upon our themes. Further research will also interpret how this developmental timeline relates to clinical cases such as congenital heart block, to aid in the early identification of developmental abnormalities.

## Methods

### DT-MRI Tissue Preparation

23 human fetal hearts with an age range from 95 to 143 days gestational age (DGA) were obtained for DT-MRI. The hearts were fixed in 4% formaldehyde before used for MRI experiments. All samples were clinically assessed and found free of any visually identifiable abnormalities and dysmorphism.

Temporary storage of tissue was in premises licensed by the 2004 Human Tissues Act (UK) and all protocols had ethical committee approval. The samples were acquired following elective termination of pregnancy, with informed written consent obtained from all subjects. All methods were carried out in accordance with relevant guidelines and regulations. All experimental protocols were approved by NHS Lothian Research and Development, the University of Edinburgh Research Governance Hope and the University of Leeds ethical committee (Study Code LREC 08/S1101/1).

The samples were obtained legally under the U.K. Abortion Act of 1967. In England, Wales and Scotland the Abortion Act 1967 allows a pregnancy to be terminated by a registered medical practitioner if the pregnancy has not exceeded its twenty-fourth week of gestation. All samples were collected after informed consent was secured by both parents (when possible) or just the mother (in the case of a single parent). All samples used in this study originated from abortions due to social reasons. Any samples from abortions performed due to medical reasons were not made available to the study. Due to this reason, it has taken us more than 3 years to collect as many samples as we could. All mothers and fetuses were being medically examined throughout their pregnancy until the time of the elective termination. No defects were seen using ultrasound in utero during gestation. All fetuses appeared morphologically normal during regular checks with the midwife and upon medical termination of pregnancy as certified by the physician. Furthermore, when MRI was performed all hearts were found structurally normal. Gestational age was measured by ultrasound scan before the procedure and confirmed by measuring foot length afterwards.

### DT-MRI Data Acquisition

The hearts were imaged in Fomblin (Fomblin Y, Sigma). The MRI acquisition protocol used here has been described in detail before^1^. For further details go to Supplementary Material S1.

### Image Processing and Tractography

We have previously described mathematical approaches to extract local fiber orientation and organization, and values of fractional anisotropy (FA; a quantitative measure of tissue organization; Supplementary Material S2) of hearts at different gestational stages^1^.

The 3D diffusion weighted images were reconstructed using DSI Studio^46^, which directly extracts eigenvectors and eigenvalues and calculates FA in a similar manner. Directional information from the eigenvalues of every voxel in 3D and values of local FA were combined to generate Direction Enhanced Color Maps (DEC) for the visualization of the ventricles and their boundaries using DSI Studio (Supplementary Figures S3 and S4).

DSI Studio also calculated the Apparent Diffusion Coefficient (ADC; a diffusion coefficient measurement suitable for heterogeneous environments such as biological tissue) by extracting the intensity values for every image voxel and their b factors using the Stejskal-Tanner model

1 $$S(b)=S(0)\ast \exp (-b\ast ADC)$$

Where S(0) is the signal intensity without diffusion weighting, S(b) is the signal intensity with the gradient and the b value.

Using DSI Studio, we characterized and extracted (in a semi-automated manner) the primary (axial) and secondary (radial) diffusion direction of the segmented ventricles (right and left) throughout gestation.

Axial diffusion (AD) is represented by the primary eigenvector **λ**_**1**_ and radial diffusion (RD) is represented by

2 $$RD=\,\frac{({{\boldsymbol{\lambda }}}_{2}+{{\boldsymbol{\lambda }}}_{3})}{2}$$

The computed cardiac fiber paths follow the local fiber orientations as calculated from the diffusion data. This method is known as tractography and it is a computational visualization method for the 3D representation of vector fields. Tractography is used to study the structure and architecture of axonal tracts in the brain of animals^31^ and humans^47^. In this study tractography was used to join voxels with close local average myofiber orientation into a path, starting from random seed point. These fiber paths, which reconstruct the global helical organization of the ventricular myocardium, are quantified by their number and lengths and represented as color-coded lines.

Tractography uses Euler’s integration algorithm^46,47^ using a step size equal to half the MRI resolution (it defines the moving distance in each tracking interval). The seed orientation was set to track the primary eigenvector field and its position was set to the subvoxel level for the detection of micro-structural features.

### Western Blotting

The Western blotting protocol used here has been described in detail elsewhere^48^. For further details on the western blotting protocol used in this study go to Supplementary Material S5.

### Data Analysis

To assess differences between myocardial development across age, linear regression analysis was performed. The sign, significance and magnitudes of correlations between age and FA, ADC and diffusivity were tested and the goodness of fit is expressed as R^2^. Protein expression was analyzed by one-way ANOVA, followed by Tukey’s post hoc tests. In all cases, α was set at <0.05. Graphs and statistics were prepared using GraphPad Prism version 6.

## Electronic supplementary material

Supplementary Material
