# Supplementary material for: Ventricular myocardium development and the role of connexins in the human fetal heart
Source: Sci Rep. 2017 Sep 25;7:12272. doi: 10.1038/s41598-017-11129-9 (PMC5612926; doi:10.1038/s41598-017-11129-9)
Supplement: Supplementary file 1 — Supplementary Material [file 41598_2017_11129_MOESM1_ESM.pdf]

Ventricular myocardium development and the role of connexins in the human fetal heart.

Eleftheria Pervolaraki[a]*, James Dachtler[b], Richard A Anderson[c], Arun V Holden[a]

[a]School of Biomedical Sciences, University of Leeds, Leeds, LS2 9JT, UK;

[b]Department of Psychology, Durham University, Durham, DH1 3LE, UK;

[c]MRC Centre for Reproductive Health, University of Edinburgh, Edinburgh, EH16 4TJ, UK.

***Address for correspondence:**
Dr Eleftheria Pervolaraki; School of Biomedical Sciences, University of Leeds, LS2 9JT, UK. fbsepe@leeds.ac.uk, +44(0) 113 34 31869.

Supplementary Material

Supplementary Material S1

DT-MRI Data Acquisition

MRI acquisition was performed in a Bruker Biospin (Ettlingen, Germany) 9.4 Tesla vertical NMR/S System with a 22mm imaging coil for Hydrogen (^1H). A three-dimensional diffusion weighted spin echo sequence was carried out at 20°C with 0.1 mm^2 resolution, echo time = 15ms, repetition time = 500ms, with 6 averages and a b value of 1000 s mm^{-2}. In each scan, diffusion weighted images were obtained in 12 directions. The average scan time was ~24 hours.

Supplementary Material S2

FA is a scalar value between zero and one that describes the degree of anisotropy of a Gaussian diffusion process; with an FA of zero being isotropic (i.e. spherical diffusion) with all three eigenvectors approximately the same (Supplementary Figure S3). FA for each voxel is calculated from the components of the diffusion tensor of that voxel within the tissue. The tissue FA (and its distribution) is estimated from the FA of all the myocardial voxels within the ventricle (left or right). We calculated the primary (λ_1), secondary (λ_2) and tertiary (λ_3) eigenvalues of the diffusion tensor for every voxel.

FA was calculated by

$$FA = \sqrt{\frac{3}{2} * \frac{(\lambda_1 - \langle\lambda\rangle)^2 + (\lambda_2 - \langle\lambda\rangle)^2 + (\lambda_3 - \langle\lambda\rangle)^2}{\lambda_1^2 + \lambda_2^2 + \lambda_3^2}}$$

$$\langle\lambda\rangle = \frac{\lambda_1 + \lambda_2 + \lambda_3}{3} \qquad [3]$$

Supplementary Material S3

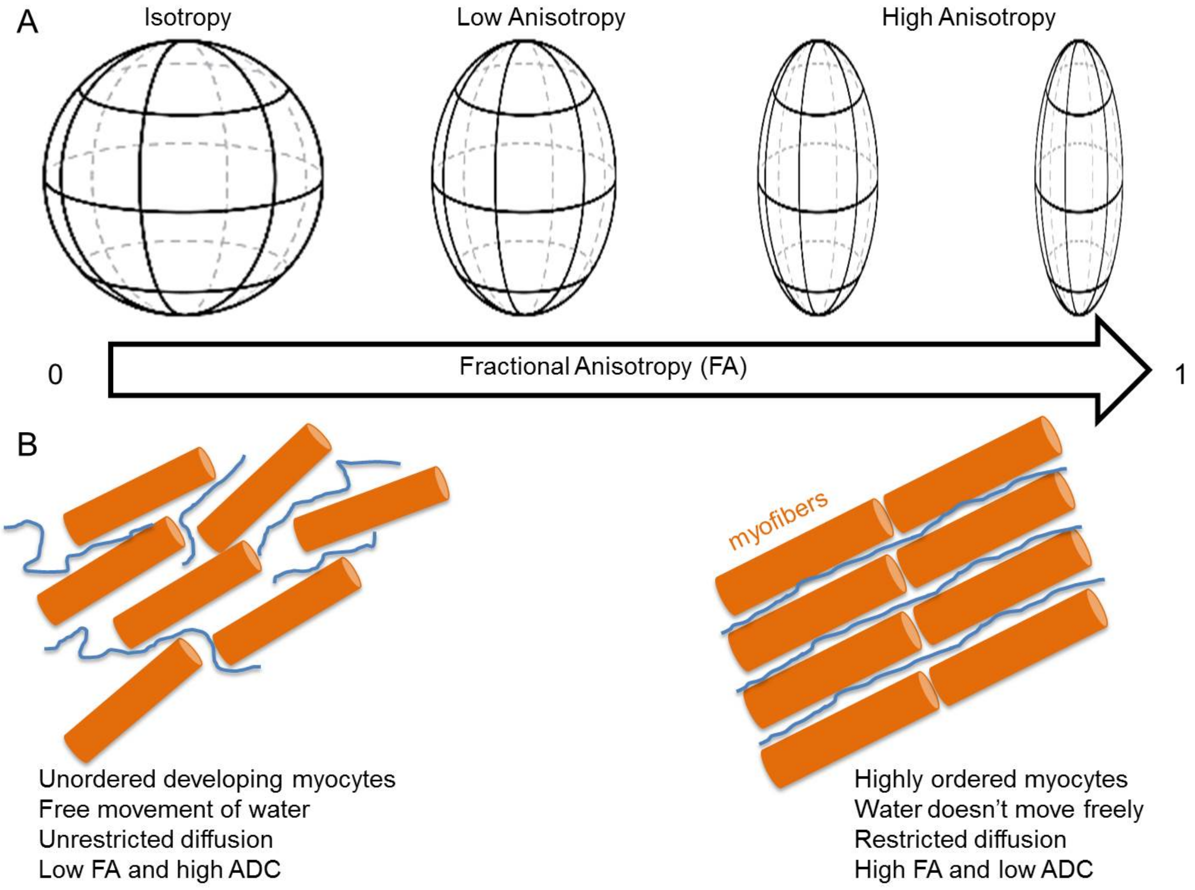

Supplementary Figure S3. Quantification of myocardial organization: (A) Fractional anisotropy defined by equation [1] increases as diffusion in one orientation (primary) becomes greater than diffusion in the other two orthogonal orientations. (B) Schematic of possible mechanisms of how tissue organization leads to changes in fractional anisotropy (FA) and apparent diffusion coefficient (ADC).

Supplementary Material S4

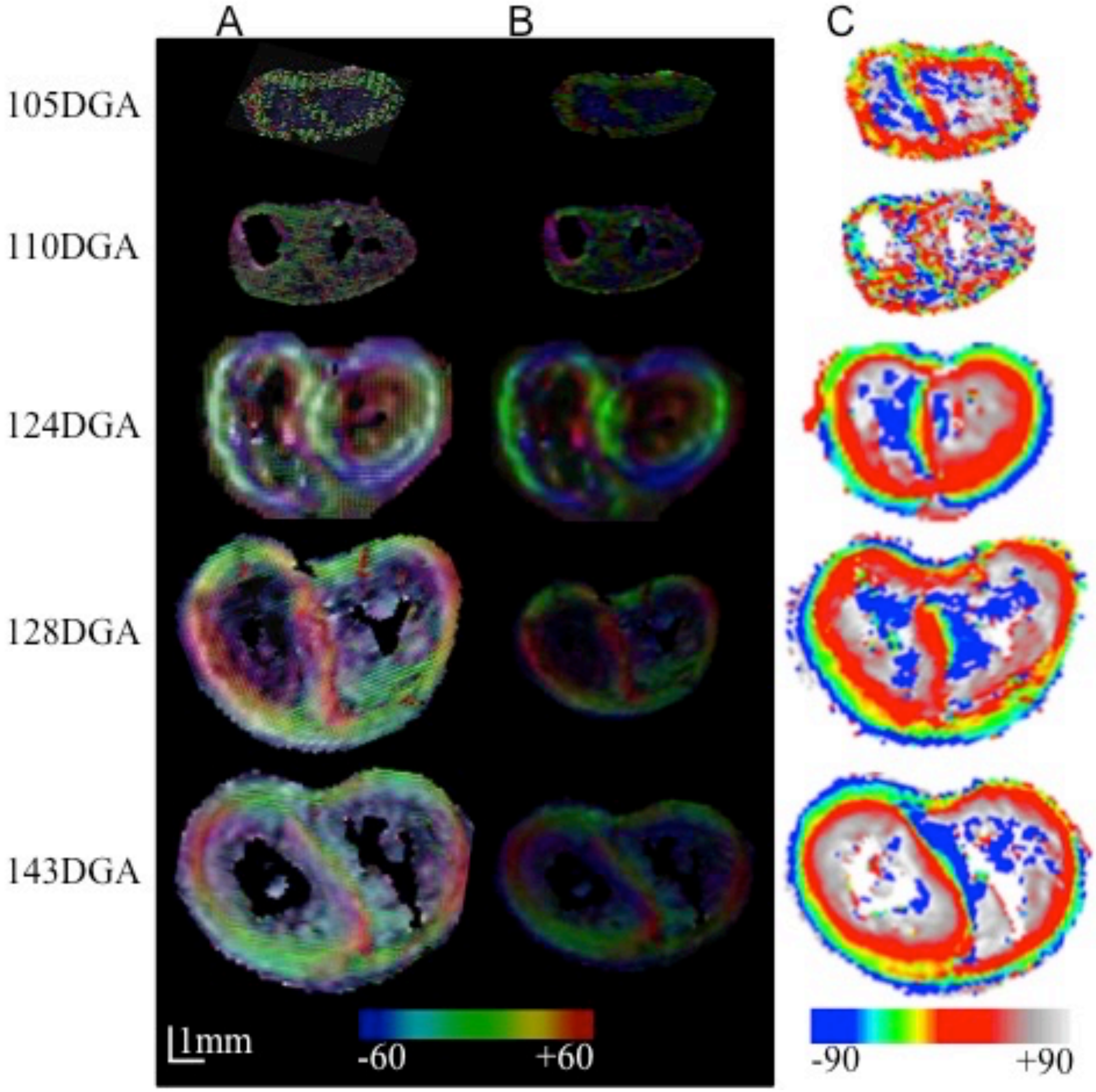

Supplementary Figure S4. Ventricular wall fiber architecture during gestation: Mid-ventricular sections of wall architecture visualized in short axis slices for fetal hearts aged 105, 110, 124, 128 and 143 DGA. (A) Local average ventricular myocyte orientation with the angle represented by a line and color map. (B) Directional encoded colormaps (DEC) used to visualize directional information of diffusion with color-coding direction obtained from the eigenvectors. (C) Myocyte orientation in the plane of the wall displayed as helix angle, obtained from the MRI derived structural datasets.

Supplementary Material S5

9 hearts ranging from 67-136 DGA (67, 70, 71, 73, 96 x2, 107, 117 and 136 DGA) were dissected, snap frozen and subsequently stored at -80°C. In a subsequent experiment, we also tested one heart aged 97 DGA. The hearts were first cut into smaller sections and then homogenized with a TissueRuptor (Qiagen, Manchester, UK) with x5 w/v RIPA Lysis Buffer containing 0.1% sodium orthovanadate, 0.1% PMSF, 0.15% protease inhibitor cocktail (sc-24948; Santa Cruz, Heidelberg, Germany) and 1× Phosphatase Inhibitor Mini (Pierce, Thermo Fisher Scientific, Paisley, UK). The homogenate was centrifuged and the protein concentration of the supernatant quantified by BCA assay (Pierce, Thermo Fisher Scientific). Samples of 20μg total protein containing Laemmli sample buffer (Bio-Rad, Hemel Hempstead, UK) with 5% β-mercaptoethanol (Thermo Fisher Scientific, Paisley, UK) were boiled at 95°C for 5min. Samples were subjected to gradient SDS–polyacrylamide gel electrophoresis (100V, ~1.5h) on polyacrylamide gels (4–20%) (Mini-PROTEAN TGX, Bio-Rad), transferred to Amersham Hybond PVDF membranes (GE Life Sciences, Buckinghamshire, UK) (120V, 75min on ice), and blocked overnight at 4°C in 2% bovine serum albumin (BSA) (Thermo Fisher Scientific) in 1× phosphate buffered saline with 0.1% Tween-20 (Thermo Fisher Scientific).

Membranes were incubated with 1:500 of the primary monoclonal antibody connexin 43 (sc-271837; Santa Cruz) or connexin 40 (sc-365107; Santa Cruz) in 2% BSA for 1h at room temperature. 1:5000 anti-mouse (sc-2371; Santa Cruz) HRP-linked secondary antibody was incubated in 2% BSA for 1h at room temperature. Bound peroxidase-conjugates were visualized using ECL Prime western blotting substrate (GE Life Sciences). Membranes were immersed in stripping buffer (Re-Blot Plus Strong, Millipore, Hertfordshire, UK) at room temperature for 20min before incubating with anti-GAPDH (sc-25778) at 1:1000, followed by 1:5000 anti-rabbit (sc-2004; Santa Cruz) HRP-linked secondary antibody (both for 1h at room temperature with 5% milk) to confirm equal loading. All western blots were repeated three times. Densitometry was performed using Image Studio (LI-COR, Cambridge, UK) with expression normalized to the GAPDH loading control. Full example blots are shown in Supplementary Figure S6.

Supplementary Material S6

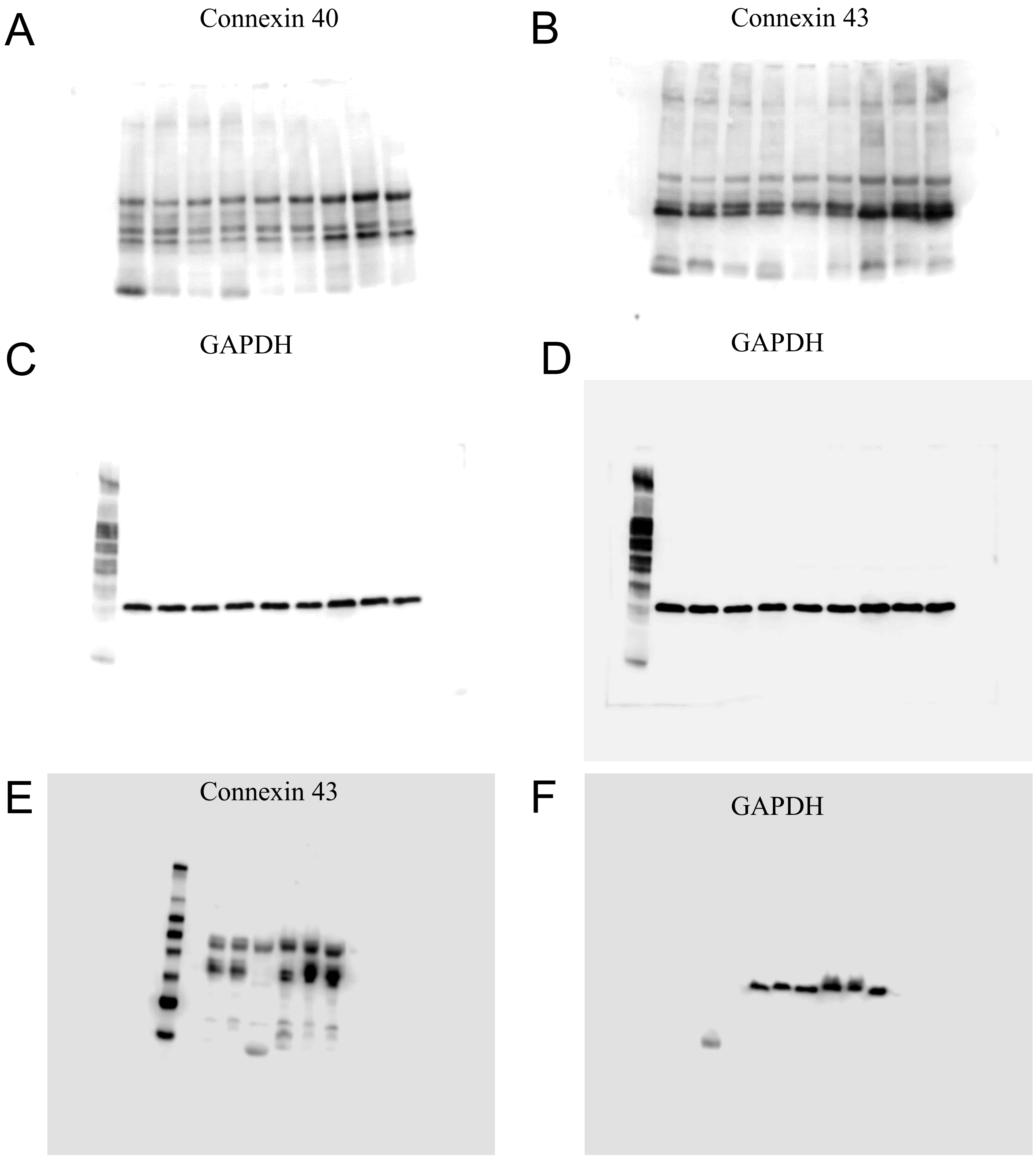

Supplementary Figure S6. Example blots of connexin western blotting: (A and C) Example full membrane blot of Cx40 and GAPDH taken from Figure 5. (B and D) Example full membrane blot of Cx43 and GAPDH taken from Figure 4. (E and F) Example full membrane blot of Cx43 and GAPDH taken from Figure 6, with Cx43 absent from the sample in lane 3 (E) but not for the corresponding loading control (F). Membranes with antibodies against connexin were stripped using stripper buffer and then probed for GAPDH as a loading control.
